# Research Progress of Electroplated Nanotwinned Copper in Microelectronic Packaging

**DOI:** 10.3390/ma16134614

**Published:** 2023-06-26

**Authors:** Ke-Xin Chen, Li-Yin Gao, Zhe Li, Rong Sun, Zhi-Quan Liu

**Affiliations:** 1 Shenzhen Institute of Advanced Electronic Materials, Shenzhen Institute of Advanced Technology, Chinese Academy of Sciences, Shenzhen 518055, Chinaly.gao@siat.ac.cn (L.-Y.G.);; 2 School of Material Science and Engineering, University of Science and Technology of China, Shenyang 110016, China; 3 Shenzhen College of Advanced Technology, University of Chinese Academy of Sciences, Shenzhen 518055, China

**Keywords:** nanotwinned copper, microelectronic packaging, electrodeposition, property, reliability

## Abstract

Copper is the most common interconnecting material in the field of microelectronic packaging, which is widely used in advanced electronic packaging technologies. However, with the trend of the miniaturization of electronic devices, the dimensions of interconnectors have decreased from hundreds of microns to tens of or even several microns, which has brought serious reliability issues. As a result, nanotwinned copper (nt-Cu) has been proposed as a potential candidate material and is being certified progressively. Firstly, the physical properties of nt-Cu have been widely studied. Notably, the higher thermal stability and oxidation resistance of the (111) texture causes nt-Cu to maintain excellent physical properties under high-temperature serving conditions. Secondly, recent works on the electrolyte and electroplating processes of nt-Cu on wafer substrates are summarized, focusing on how to reduce the thickness of the transition layer, improve the twin density, and achieve complicated pattern filling. Thirdly, nt-Cu can effectively eliminate Kirkendall voids when it serves as UBM or a CuP. Additionally, the high (111) texture can control the preferred orientation of interfacial intermetallic compounds (IMCs) at the Cu–Sn interface, which should be helpful to improve the reliability of solder joints. nt-Cu has superior electromigration resistance and antithermal cycling ability compared to ordinary copper RDLs and TSVs. Above all, nt-Cu has attracted much attention in the field of microelectronic packaging in recent years. The preparation–performance–reliability interrelationship of nt-Cu is summarized and displayed in this paper, which provides a solid theoretical basis for its practical applications.

## 1. Introduction

With the emergence of integrated circuit (IC) technologies, the development of electronic devices is altering from day to day. Moore’s law proposed in 1965 is no longer suitable for IC development at present. Advanced packaging technologies are the key pathway to achieving “More than Moore”. Packaging methods have evolved from plating through hole (PTH), the pin grid array (PGA), and the ball grid array (BGA) in the last century to the multichip module (MCM) and the multidimensional structure. Since the start of the 21st century, new information-processing systems with dramatically improved energy efficiency and performance are partly based on advanced packaging technologies, such as flip-chip and wafer-level packaging. The interconnection form has evolved from the initial wire bonding to under-bump metallization (UBM), copper pillars (CuPs), redistribution layers (RDLs), through-silicon vias (TSVs), etc.

**Figure 1:**
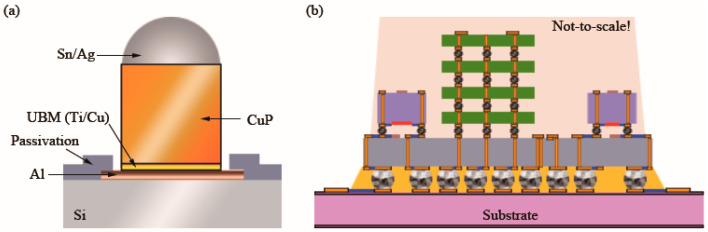
Schematic diagram of the cross-section of (**a**) a CuP and UBM on a wafer and (**b**) a heterogeneous integration of various chips on a TSV (orange perpendicular line) interposer with RDLs (in blue) [2].

UBM is an indispensable technology for the purpose of soldering metal pads and tin balls, as shown in Figure 1a. The functions of UBM include the following: (a) a bonding layer for interconnection; (b) preventing atomic diffusion between tin balls and a bottom material; and (c) binding a layer of dielectric material and a metal layer. As the interconnection density increased, CuP technology appeared. Increasing the volume (thickness) of rigid copper and decreasing the volume of solder can decrease the pitch of a solder joint and increase the diffusion barrier ability. RDLs can reconfigure or redistribute pads from the perimeter footprint to alternative locations on a chip, enabling wafer bumping as shown in Figure 1b [1]. Therefore, the use of RDLs allows the utilization of a greater area of the chip and enables the use of simple, less expensive substrates. TSVs realize the electrical interconnection between chips in the vertical direction, as shown in Figure 1b. As a result, TSV-based 3D IC technology can dramatically reduce the volume of electronic devices.

**Figure 2:**
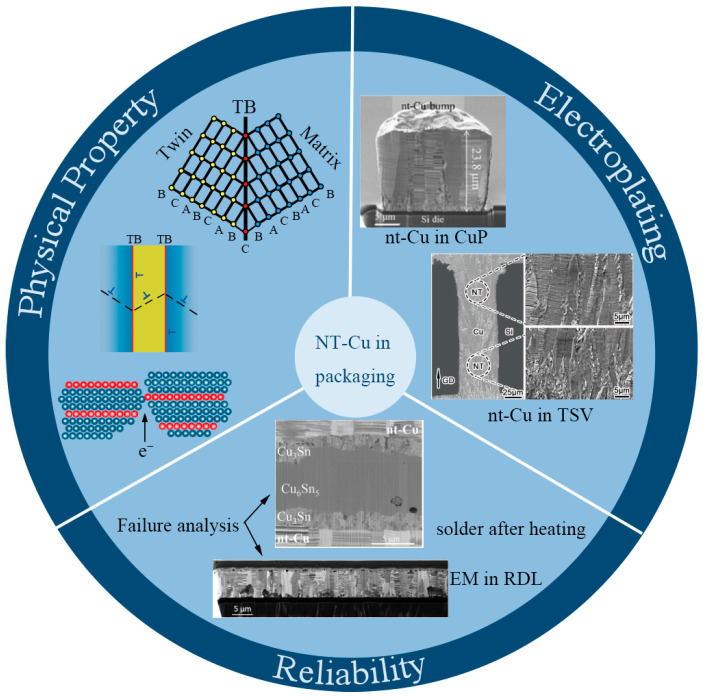
Property–electroplating–reliability interrelationship of nt-Cu materials [21,22,23,24,25,26]. TB represents the twin boundary. A, B and C represent atomic arrangements. “T” symbols represent dislocations. Red dots represent the atoms of TB. Other color dots represent the atoms of matrix.

Meanwhile, the decreasing size and pitch of RDLs, UBM, CuPs, and TSVs put a great challenge on the reliability of interconnection technologies. Although interconnection materials have changed from traditional aluminum (Al) and its alloys to current, mainstream copper (Cu) [3], after IC technology entered the 32 nm node, an interconnector’s maximum effective current-carrying density is far away from the demand. Reliability problems, including voids, electromigration (EM), and the dielectric breakdown of copper interconnections, hinder the further development of ICs [4]. In addition, the effect of EM is becoming increasingly prominent. In recent years, researchers have developed several potential interconnection technologies, such as carbon-based interconnection technology [5], optical interconnection technology [6], etc. In this case, nanotwinned copper (nt-Cu) interconnections have also been raised as a modified version of copper interconnection, and nt-Cu has been widely reported as one of the potential materials to cater to the reliability requirement of interconnections [7,8,9,10]. In 2004, K. Lu’s group found that nt-Cu exhibited ultrahigh strength and high electrical conductivity [11]. Followed closely by other researchers, some excellent physical properties of nt-Cu have also been discovered [12,13,14,15]. Since then, nt-Cu has been continuously studied as a vigorous substitute for common Cu in different fields, such as applications for aggressive radiation environments [16], a growth substrate for boron nitride [17], electrocatalytic water splitting [18], and an electrode current collector in lithium-ion batteries [19,20]. In 2012, Chih Chen’s group [21] utilized nt-Cu as a UBM for the unidirectional growth of solder bumps, signifying a landmark event of nt-Cu applied in electronic packaging interconnecting materials. Up to now, there has been a great quantity of research on nt-Cu in microelectronic packaging technologies, focusing on preparation methods, microstructure characterization, physical property optimization, and reliability evaluations, as shown in Figure 2. 

Compared with traditional commercial Cu, nt-Cu has achieved an overall superior performance in advanced packaging. Therefore, in this paper, the physical properties, electroplating fabrication process, and reliability performance in microelectronic packaging are reviewed and commented on, and the property–electroplating–reliability interrelationship is further discussed.

## 2. Microstructure and Property Optimization of nt-Cu

Distinctive properties of nt-Cu originate from a special atomic arrangement, so its microstructure is firstly introduced before the introduction of properties. Then, the role of nanotwins in the mechanical and electrical properties, thermal stability, and oxidation resistance are discussed. The distinctive properties lay a solid base for nt-Cu application in the field of electronic packaging.

### 2.1. Lattice and Atomic Arrangement of nt-Cu

**Figure 3:**
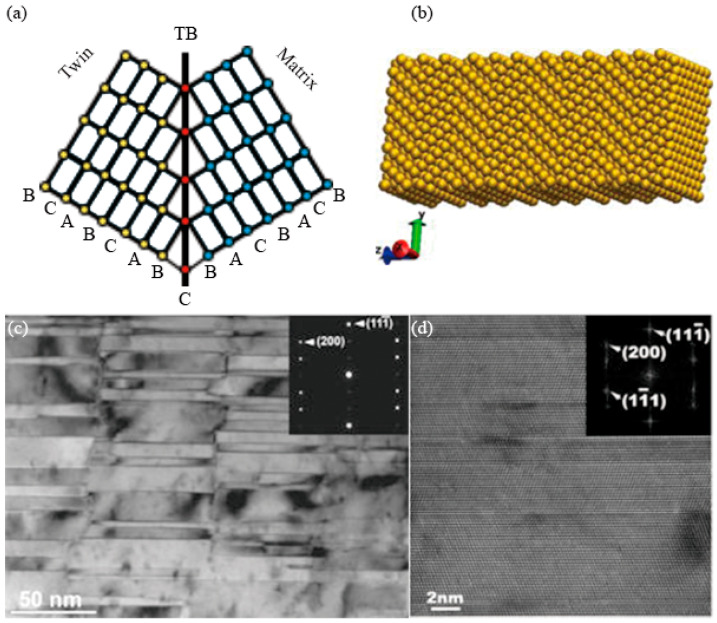
Schematic illustrations of (**a**) crystal lattices of grains with a twin boundary [22] (A, B and C represent atomic arrangements. Red dots represent atoms which composed of TB. Other colored areas represent there is no TB arrangement.) and (**b**) the nanotwinned structure of an nt-Cu supercell [28]. (**c**) TEM image of Cu with twin planes oriented vertical to the growth direction. Inserted SAD pattern confirms typical twin pattern [29]. (**d**) HRTEM micrograph of nt-Cu showing that the planar defects are growth twins with {111} interfaces. The inserted fast Fourier transform of the image shows spot splitting across the {111} twin interfaces [30].

During the growth of Cu grains, atoms located on the (111) plane have two sequences, i.e., a normal sequence (-A-B-C-A-B-C-A-B-C-A-B-) and a twin fault sequence (-A-B-C-A-B-C-B-A-C-B-A-). They are also called normal nucleation and twinned nucleation, respectively [27]. The -B-C-B- sequence is equivalent to a stacking fault, and the grains (or two parts of one grain) on both sides of the C-axis (also called the coherent twin boundary, CTB) show mirror symmetry, as shown in Figure 3a. The structure of an nt-Cu supercell is shown in Figure 3b [28]. The difference between nt-Cu and cg-Cu is that there can be seen a high density of nanotwins of the {111}/[112] type growing in grains of nt-Cu, and the twin boundaries (TBs) are perfectly coherent [11]. The CTBs are clear lines, as shown in Figure 3c, and they are atomically sharp, as observed in the high-resolution transmission electron microscopy (TEM) images of Figure 3d [29,30]. 

The CTB interface possesses low interfacial energy (approximately 1/10 that of ordinary grain boundaries), which is thermodynamically stable. Thus, the stable CTB is the key factor influencing the physical properties of nt-Cu. However, there are some deviations between the real state of nt-Cu prepared films and the ideal state, as stated above. Firstly, nanotwin boundaries are partly incoherent and possess higher interfacial energy (approximately 1/2 that of ordinary grain boundaries). Secondly, the grain size and grain boundaries interact with twin boundaries to produce triple or quadruple nodes. Thirdly, the impurities in a film can also affect the properties. 

In fact, studies on the physical properties of nt-Cu films have been extensively reported, including hardness, strength, elongation, thermal conductivity, electric conductivity, etc. Moreover, the experimental results vary at high and low levels due to the difference in preparation methods and microstructures (including the transition layer, grain size, twin density, etc.).

### 2.2. Mechanical Properties of nt-Cu

**Figure 4:**
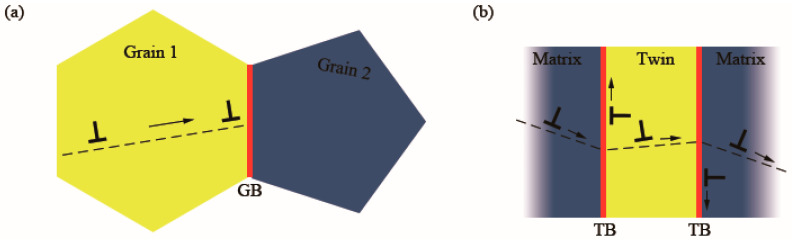
Schematic illustrations of the strengthening method in (**a**) common grains and (**b**) grains with TBs [22]. Red lines represent the GBs and TBs. Other colored areas represent homo-orientation lattices. “T” symbols represent dislocations. Arrows indicate the direction of dislocation movement.

Solid alloying, cold working, and grain refinement are common strengthening methods of metals. However, they all have side effects on ductility and electric conductivity. According to the traditional Hall–Petch relationship, strength is inversely proportional to the square root of grain size due to the difficulty of dislocations transferring from one grain to the next through the barriers of incoherent internal grain boundaries, as shown in Figure 4a [22]. However, the accumulation of dislocations in grains brings a significant increase in location stress. The stress cannot be relaxed in the tensile strain-to-failure test, so the brittleness of fcc metals increases and the ductility decreases. The maximum strain before fracture decreases, and the formation of necking and cracks are accelerated after yielding [31]. Different from incoherent boundaries with high interfacial energy, coherent internal grain boundaries, such as twin boundaries with low interfacial energy, not only can effectively block dislocation movement but also can relax local stress, as shown in Figure 4b. When the twin spacing is at the micrometer level, the annealing TB displays comparable strength to a conventional grain boundary (GB), and both of them conform to the Hall–Petch (H–P) relation [32]. The atoms of CTBs are shared by grains on the sides without atomic misfits. This kind of boundary can block dislocation motion and store dislocations. Deformation in the tensile strain-to-failure test is influenced by the interaction of dislocations and CTBs. Some categories of dislocation can cross-slip on or be transmitted across CTBs, which can effectively relax the local stress after yielding. Therefore, mechanical strength and ductility are both promoted in films with CTBs. 

**Figure 5:**
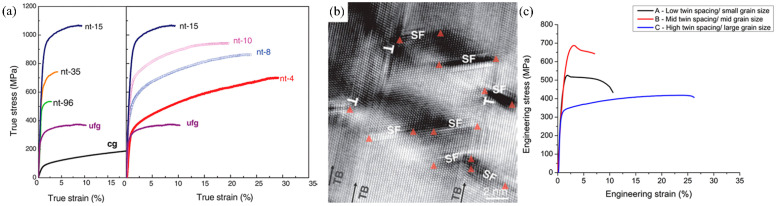
(**a**) Uniaxial tensile true stress–true strain curves for nt-Cu samples with different thicknesses from 4 to 96 nm (marked near the curve) compared with ufg-Cu and cg-Cu. (**b**) The arrangement of stacking faults at TBs within the lamellae in the nt-4 sample. Triangles represent Shockley partial dislocations associated with stacking faults; ⊥ represents the partials with their Burgers vector parallel to the TB plane [33]. (**c**) Engineering stress–engineering strain curves of three types of <111>-oriented nt-Cu with different twin spacing and grain size [34].

K. Lu’s group [11] first found that Cu film with a high density of nanotwins possessed a tensile strength about 10 times higher than that of conventional cg-Cu. An nt-Cu film with a 15 nm twin thickness (grain sizes between 100 nm and 1 μm, with an average value of about 400 nm) had stronger strength than cg-Cu and ultrafine-grained Cu (ufg-Cu), as shown in Figure 5a [33]. Dislocations could be seen inside the thick lamellae in Cu films with low densities of twins, which were analogous to the cg-Cu films. Additionally, dislocations were in the vicinity of TBs for a film with a high TB density. Many Shockley partials could be detected at TBs in high-twin-density film, as shown in Figure 5b, and they made TBs rougher than before the tensile test. This phenomenon proved that a single dislocation could penetrate a TB if the twin spacing was too thin for dislocation pile-up, and a higher external stress was required. This was the reason that the spacing of twins influenced the strength of films [33]. However, Chih Chen’s group [34] further considered the mutual effect between grain size and twin density and found that a Cu film with medium grain size instead of the largest or smallest grain size exhibited the best engineering stress, as shown in Figure 5c, which also meant the traditional strengthening theories could not explain columnar <111>-oriented nt-Cu totally. In addition, Zhi-Quan Liu’s group [35] demonstrated the effect of the transition layer on the strength of nt-Cu films. A transition layer formed between the seed layer and nanotwin region possessed less twin density and, the smaller the proportion of the transition layer, the stronger the tensile strength of the whole nt-Cu film obtained.

Grain size, thickness of twin lamella, and distribution of twins significantly influence the mechanical properties of nt-Cu. In any case, nt-Cu exhibits superior mechanical properties over those of common cg-Cu. Further optimization directions may lie in microstructure regularity enhancement and impurity concentration reduction.

### 2.3. Electrical Properties of nt-Cu

GBs can capture or scatter electrons according to previous studies [11]. Therefore, the conductivity of Cu decreases if inducing GBs in a film. However, K. Lu’s group [11] found that an nt-Cu film had a similar conductivity in comparison with a cg-Cu film. Kim and coworkers [36] measured the independent resistance of different types of GBs through four-probe scanning tunneling microscopy (STM) directly. The degree of GBs scattering electrons depended on the symmetry of grains, and the resistivity of Σ3 (TB) was calculated at about 0.202 × 10^−12^ Ω/cm^2^, which was from one to two orders of magnitude lower than that of other random boundaries. O. Angeroglu and coworkers [29] prepared epitaxial Cu with {111} nanotwins on single-crystal Cu substrates and found that these epitaxial nt-Cu films exhibited better conductivity than columnar-grain nt-Cu films. L. Yue and coworkers [37] simulated defect generation in Cu films with different textures during the sliding wear process. The least dislocation accumulation was in nt-Cu, which meant a minimal impact on the conductivity of Cu films with nanotwins. Notably, impurity was another aspect needing to be considered in order to maintain high conductivity.

**Figure 6:**
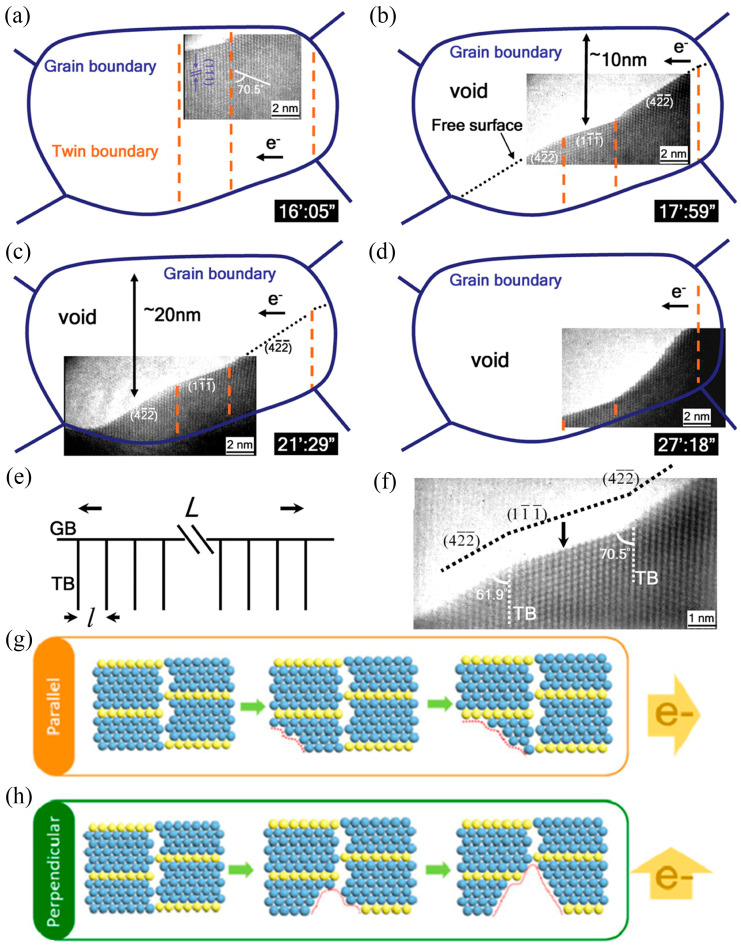
(**a**–**d**) Diagrams of the GBs and TBs of ($01\bar{1}$)-oriented Cu grains at different stages during electric current stressing with the HRTEM image captured given in the inset. (**e**) Cartoon of an nt-Cu grain of L dimensions with an average twin lamella width of *l*. (**f**) HRTEM image of a grain edge revealing the direction of EM-induced voiding [38]. (**g**,**h**) Schematic illustrations of specimens with electron flows perpendicular and parallel to the TBs, respectively [23].

Superior electromigration (EM) resistance is also necessary for interconnected materials, which determines the reliability of electronic devices. Chien-Neng Liao’s group [38,39] observed the EM-induced atomic diffusion of TBs near room temperature using in situ TEM. The triple point of the CTBs and GBs could reduce the EM-induced atomic transport, as shown in Figure 6a–f. The EM time at the triple point was an order of magnitude lower than other locations. This could be attributed to the longer incubation time of new steps at the triple point, which slowed down the atomic transmission. In 2021, Fang-Chun Shen and coworkers [23] observed the interaction of electron flow and TBs using in situ HRTEM and found that the generation of voids could be suppressed by columnar nanotwins, and as a result, the expansion rate of the void was an order of magnitude lower than that of cg-Cu. The specimen with an electron flow direction perpendicular to the TBs showed better performance for EM resistance than that with a flow parallel to the TBs. As shown in Figure 6g,h, different atomic migration behaviors occurred when the electrons flowed across Cu films with different textures.

### 2.4. Thermal Stability and Corrosion and Oxidation Resistance of nt-Cu

**Figure 7:**
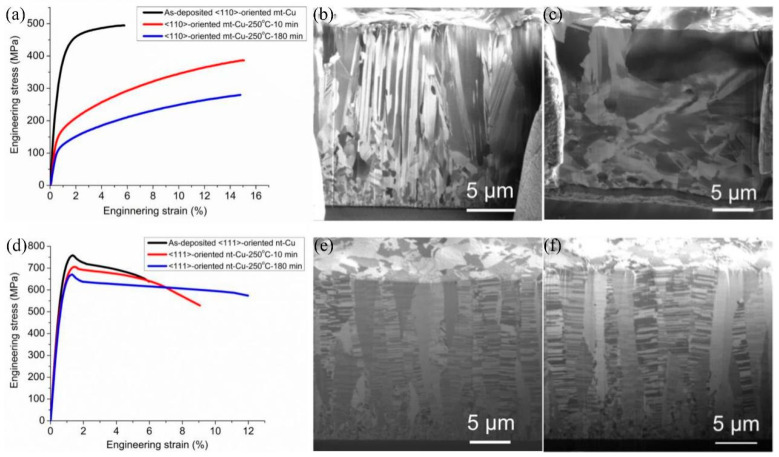
The stress–strain curves of (**a**) <110>-oriented mt-Cu foils before and after annealing at 250 °C for 10 min and 180 min. Cross-sectional ion images of mt-Cu (**b**) before and (**c**) after annealing at 250 °C for 180 min. (**d**) The stress–strain curves of <111>-oriented nt-Cu foils before and after annealing at 250 °C for 10 min and 180 min. Cross-sectional ion images of nt-Cu (**e**) before and (**f**) after annealing at 250 °C for 180 min [42].

In the past, the thermal stability of nanostructured metals has been improved by alloying to reduce the recrystallization kinetics. In 2008, O. Anderoglu and coworkers [40] investigated the thermal stability of nt-Cu films under the condition of high-vacuum annealing up to 800 °C for 1 h. The results proved that a low-angle CTB arrangement in the films could exhibit better thermal stability than monolithic nanocrystals with high-angle GBs. In 2011, C. Saldana and coworkers [41] argued that thermal instability may link to excess vacancy concentration and that reducing vacancy supersaturation could reduce the driving force and mobility for the evolution of a microstructure and improve the thermal stability of Cu. A smaller vacancy supersaturation was represented in densely twinned samples, and this may be the reason why nt-Cu exhibited better thermal stability. In 2020, Chih Chen’s group [42] compared the thermal stability of <111>-oriented nt-Cu and <110>-oriented microtwinned Cu (mt-Cu), as shown in Figure 7a–f. It can be observed that the mechanical strength was drastically reduced, and grains changed after annealing in mt-Cu foils. However, the <111>-oriented nt-Cu film exhibited high thermal stability and high mechanical strength after annealing at 250 °C. Zhi-Quan Liu’s group compared the thermal stability of nt-Cu with three other Cu films electroplated with commercial electrolytes and found that the nt-Cu films had better thermal stability than common Cu films annealed from room temperature to 400 °C. The importance of thermal stability for nt-Cu applied in microelectronic packaging is discussed in detail in the last chapter on reliability.

Ming-Hui Zhang [43] also investigated the recrystallization behavior of Cu films with different microstructures and found that the grain growth of polycrystalline Cu was controlled by the Ostwald ripening mechanism during aging at 400 °C. However, nt-Cu with high-density TBs could form stable structures, and the TBs could store large stress to facilitate anisotropic grain growth during aging. Moreover, the thickness of transition layers also influenced the grain size of nt-Cu after annealing. Kuan-Ju Chen and coworkers [44] fabricated nt-Cu with different transition layers by regulating reverse current parameters. According to the results after annealing, it was speculated that the critical factor for the grain size of <100>-oriented grains was mainly related to the thickness of the transition layer because the thick transition layer provided more than <100> nucleation sites for recrystallization. A thinner transition layer in nt-Cu could lead to a larger grain size of <100>-oriented grains after annealing.

Nt-Cu also has a better performance in corrosion resistance than that of common polycrystalline Cu under high-temperature and high-humidity conditions [10]. In 2015, Chien-Neng Liao’s group [45] investigated the effect of TB spacing on the surface chemical reactivity of Cu nanowires (NWs) and found that the nt-Cu NWs exhibited a surface structure with low atomic step density when the TB spacing was less than 10 nm. The atomic step density decreased with the decrease in TB spacing in the surface, leading to high chemical reactivity and excellent corrosion resistance. The conclusion was that the structure with high nanotwin density and thinner TB spacing on the surface had superior corrosion resistance. It may also be attributed to the reason of the superior oxidation resistance of nt-Cu. Notably, the higher thermal stability and high corrosion and oxidation resistance of the (111) texture cause the excellent physical properties of nt-Cu to be maintained under high-temperature serving conditions.

## 3. Electroplating nt-Cu in the Field of Microelectronic Packaging

Many methods can be used to produce TBs in Cu foils, such as dynamic plastic deformation [46], phase transformation [11], annealing [47], magnetron sputtering [29,30,48,49], and electroplating. Among them, electroplating is the most common and vital technology to realize electrical interconnection due to its simple operation (room temperature, normal pressure) and low cost. Therefore, the electroplating method of nt-Cu also possesses the highest compatibility with microelectronic packaging applications. We only review the development and progress of the electroplating preparation method in this paper. The electrolyte and electroplating process optimization of nt-Cu films upon wafer substrates has been widely studied. They are mostly aimed at reducing the thickness of transition layers, reducing twin lamellar spacing, improving smoothness, and achieving complicated pattern fillings, including different aspect ratio vias and fine-pitch RDLs.

### 3.1. Electroplating Process of nt-Cu

It is well known that the process of electroplating can be roughly divided into pulse electrodeposition (PED) and direct current (DC) electrodeposition depending on the difference in the electric field. At first, the pulse electric field was considered to be the key method to generate nanotwins. However, nt-Cu was also obtained during a DC electroplating process. Therefore, the formation mechanism of nanotwins during the electroplating process remains a controversial topic. In our opinion, twin growth can be controlled by aspects of the electric field, chemical field, and hydrodynamics field. 

#### 3.1.1. Effect of Pulse Parameters

It was regarded that the pulse electric field should be one of the methods to generate nanotwins [50]. From a thermodynamic point of view [28,51], a pulse electric field with a high peak current density in its on time (t_on_) and no current density in its off time (t_off_), alternatively, can achieve rapid deposition and renucleation, respectively. Cu^2+^ ions in the vicinity of the electrode are depleted greatly during t_on_, and the nucleation sites increase accordingly. During t_off_, the Cu^2+^ ions can be replenished, and recrystallization substitutes for grain growth, which may facilitate twin formation.

In detail, Di Xu and coworkers [28] studied the influences of stress/strain on the formation of nanotwins through first-principle calculations and proposed that strain-relaxed nanotwins formed during the recrystallization process when biaxial stress was applied and the strain was large enough in a Cu film. Therefore, stress generation and relaxation occurred during the pulse on time and off time, respectively, forming nanotwins in the PED process. The in situ stress was measured during the PED process of Cu films and showed an increase in tensile stress to about 400 MPa during t_on_ = 0.1 s and stress relaxation during t_off_ = 9.9 s [51]. This result demonstrated that stress relaxation during t_off_ was the consequence of nanotwin formation. Xiaofei Zhan and coworkers [52] observed a spiral feature of a hexagonal pyramid on the top of nt-Cu films, and the formation of periodic twin structures could be the result of the screw-dislocation-induced growth, which could also be seen in the twinned SiC nanowires [53]. An indirect approach was proposed to observe the microstructural evolution in the PED process. Man-made layers were introduced as marking sites, which could reflect the interface morphology and position at particular moments. The re-entrant corner (70.53°) was utilized to explain the constant growth of a tapered interface along the <111> direction and the formation of high-density twins. The re-entrant corner had four nearest neighbors to function as a preferential growth site, and it could appear through the growth of (111) planes. A periodic twin structure could form during the repetition of appearance and disappearance of the re-entrant corner, which functioned as a self-perpetuating step source. In addition, some defects could provide a step source on the (111) nt-Cu formation surface in some opinions [54]. 

**Figure 8:**
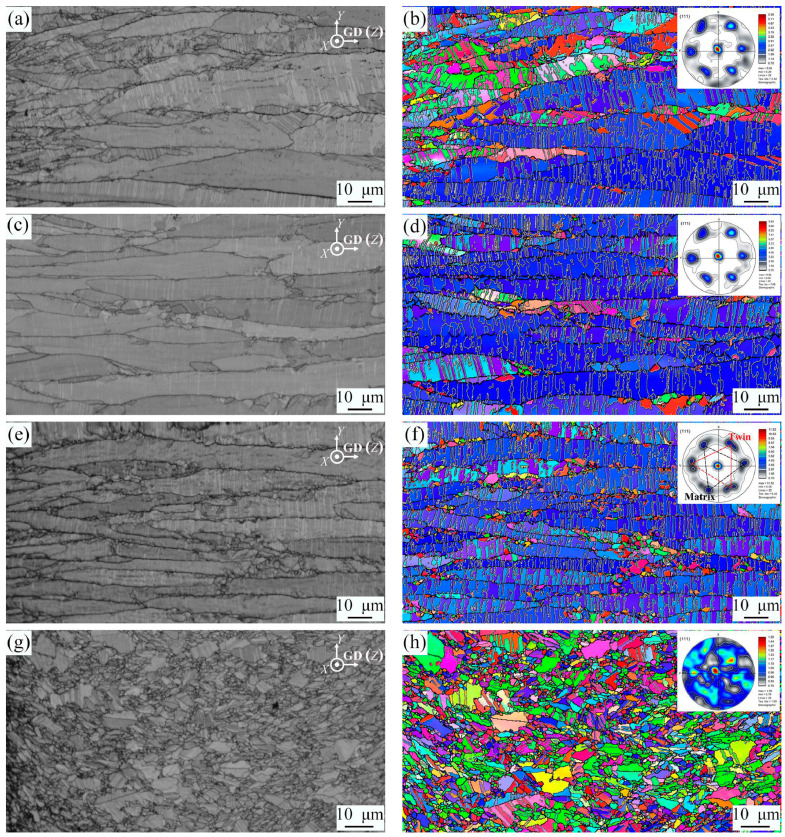
EBSD pattern quality and corresponding orientation distribution images of as-deposited Cu with different f values (Hz): (**a**,**b**) 10, (**c**,**d**) 100, (**e**,**f**) 1000, and (**g**,**h**) 2000 [52]. (For interpretation of references to color in this figure, the reader is referred to the web version of this article).

The detailed influences of duty ratio and frequency are also discussed as follows. The duty ratio (γ) refers to the ratio of on time to the sum of on time and off time (γ = t_on_/t_on_ + t_off_). The ton needs to be short enough with a high current density for Cu^2+^ ion deposition, while the t_off_ of the PED process has to be as long as necessary to enable ion transport into the depleted vicinity of an electrode and as short as possible in order to minimize grain growth [50]. Usually, the γ is about 5% or greater in the PED process [55], and a higher t_on_ current is required in a low γ PED process to obtain the same deposition rate as that of a DC process. In 2022, Yu-Xi Wang and coworkers [56] obtained a series of vertical nt-Cu films by changing t_off_ and found that, the longer the t_off_ (smaller γ), the denser the twin density obtained. It was reported that the thickness of twin lamellae decreased when the frequency of PED increased, while the γ was kept at 30%, as shown in Figure 8 [52]. There are also some works demonstrating that twin density may be promoted with a higher frequency, as previously inferred [57]. 

#### 3.1.2. Effects of Other Influencing Factors

**Figure 9:**
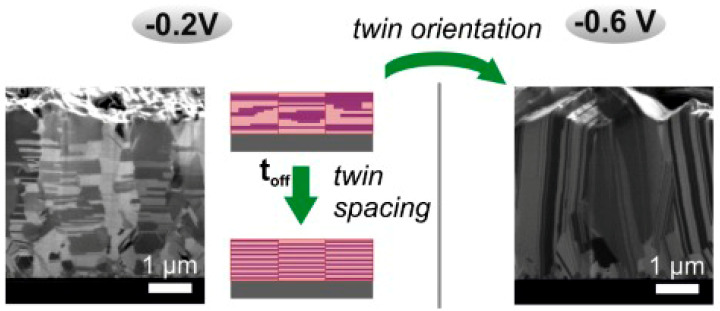
Schematic diagram of orientation-controlled nt-Cu obtained by changing electroplating potential [59].

The roles of influencing factors, such as current density and temperature, are discussed in both PED and DC electrodeposition, too. Current density can determine the existence and orientation of twins on a premise where the concentration of Cu^2+^ ions is constant for either PED or DC electrodeposition. According to the Winand diagram, a larger cathode current density, a lower concentration of Cu^2+^ ions, and higher inhibition intensity are conducive to the formation of twins. In addition, Cu deposits transform from the basis-oriented reproduction (BR) type to the field-oriented texture (FT) type, of which (111) nt-Cu can be a special form [58]. The growth potential in the PED process can alter the twin orientation from the horizontal to the vertical direction. In detail, Madoka Hasegawa and coworkers [59] prepared horizontal nt-Cu at −0.2 V vs. a saturated calomel electrode (SCE) and vertical nt-Cu at −0.6 V vs. an SCE with the PFD process, as shown in Figure 9.

The temperature also influences the formation of twins [50]. The variation in electrodeposition temperature can control the diffusion of ions and nucleation. A high adsorption–desorption rate and grain growth rate can be achieved at a high temperature, which makes it oppose the formation of twins. In 2014, Chien-Neng Liao’s group [27] fabricated dense nt-Cu nanowire arrays through the PED process at low temperatures (−1 °C). The temperature dominated the existence of twins without the influence of current density.

In addition, Chih Chen’s group [60] induced nt-Cu produced by DC electroplating with a high current density and high stirring speeds. The (111)-oriented nt-Cu was fabricated with more than 20 μm thick dense nanotwins at 20–100 mA/cm^2^ with 400–1500 rpm. The shear force could work at the cathode surface to form twins during DC electroplating with a high stirring rate. However, an increased stirring rate cannot guarantee the formation of twins, and the role of additives in electrolytes should be considered. In particular, nanotwins were easily formed when gelatin was added into the electrolytes. The inhibition intensity, which is controlled by additives, is discussed from the aspect of the chemical field in Section 3.2.

To summarize, the growth of twins can be driven kinetically when grain nucleation is facilitated and the growth of crystallites is strongly impeded [50]. The pulse electric field may be the most important parameter for nanotwin nucleation in the PED process while, for the DC process, stirring and electrolyte components may be more important for nanotwin nucleation. Other parameters, such as current density and temperature, have significant impacts on twin orientation, distribution, and density.

### 3.2. Electroplating Solutions of nt-Cu

**Figure 10:**
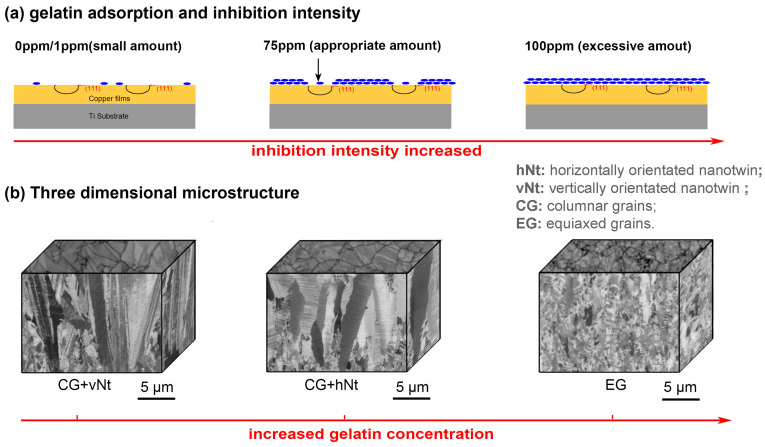
Schematic diagram of (**a**) gelatin adsorption on a cathodic surface and (**b**) construction of a three-dimensional microstructural model with increasing concentration of gelatin [62].

In the beginning, the simple electrolyte of CuSO_4_ with some acidity could obtain nt-Cu films through PED processes [11]. Later, researchers found that some organic molecules could promote nanotwin growth during the DC electroplating process. Yu Bai and coworkers [61] fabricated vertical nt-Cu with a (220) texture through DC electroplating at a high current density of about 3–5 A/dm^2^ (ASD) with commercial additives. In 2022, Zhong-Guo Li and coworkers [62] demonstrated that gelatin could work as an inhibitor in the DC electrodeposition process with electrochemical measurements. The inhibition intensity of gelatin was proportional to its concentration. The selective adsorption of gelatin on the (111) surface of Cu resulted in the transformation of a Cu film from the (110)-preferred vertical nt-Cu (vnt-Cu) to the (111)-preferred horizontal nt-Cu (hnt-Cu). When the content of gelatin exceeded the saturated concentration, the texture of the Cu film changed from a columnar crystal to an equiaxed crystal. This work considered the detailed crystal orientation and direction of nanotwins, which extended the original Winand diagram. The newly established gelatin-controlled Winand (GCW) growth model is shown in Figure 10. Similarly, it was also reported by Chia-Hung Lee and coworkers [63] that the density of nanotwins could be controlled by changing the gelatin concentration.

#### 3.2.1. Effect of Acidity

The acidity of an electrolyte also affects the growth of twins. Chih Chen’s group [64] reported that the addition of H_2_SO_4_ ranging from 50 to 110 g/L in CuSO_4_-based electrolytes could control the growth of twins under different current densities. The Cu^2+^ worked as the main conductive ion when no acid was in the electrolyte, while H^+^ replaced this role when acid was added. In this case, the diffusion of Cu^2+^ was influenced by concentration gradients. Therefore, sulfuric acid could inhibit the growth of columnar nt-Cu. Zhi-Quan Liu’s group [65] also studied the effect of acidity on the microstructure of Cu films. An increased concentration of sulfuric acid promoted the formation of twins, which further led to a thinner transition layer. In addition, the high acid concentration could transform the columnar grains into equiaxed grains and decrease the nanotwin density. The following year, this group [66] also reported that the internal texture of Cu films could be controlled by varying the acid concentration in the electroplating bath. The cathodic overpotential theory was put forward where the overpotential could provide the energy for the nucleation of twins, stemming from the adsorption of hydrogen ions on the cathode.

#### 3.2.2. Effect of Additives

Adding additives is the key to achieving perfect pattern filling. However, the role of additives in the electrolyte may be detrimental to the growth nt-Cu. Therefore, how to balance superb filling ability and a high density of nanotwins is the primary challenge to using nt-Cu materials in the field of microelectronic packaging.

As a general rule, accelerators are small molecules with sulfur atoms and negative electrification in the plating bath. In 2022, Yu-Xi Wang and coworkers [67] controlled the concentration of MPS to obtain dense hnt-Cu. Damascene vias with an aspect ratio of 1.4:1.4 (unit: μm:μm) could be filled without voids using co-additives containing PEG and 40 ppm of MPS.

As the most confidential ingredient in commercial electroplating solutions, leveler plays a vital role in filling. Usually, the molecules of leveler provide negative electrification in a bath containing nitrogen atoms. Levelers can be adsorbed on a protrusion area with a high current density to achieve a leveling effect. Common levelers include JGB, diazine black (DB), dodecyl trimethyl ammonium chloride (DTAC), etc. Jing Huang and coworkers [68] tried to improve filling performance by adding additives. Methylene blue was used as an additive at first during the DC electroplating process. A high-density nanotwin structure was formed from bottom to top when the concentration of methylene blue was 2 mg/L. In another case [69], when cetyltrimethylammonium bromide (CTAB) was added into a nanotwinned electrolyte, the cathodic polarization was significantly enhanced, which meant that there were more electrons in the cathode, and nanotwins appearing with the deposition of Cu were inhibited. Later, Jing Huang and coworkers [70] also found that the presence of Janus green B (JGB) also could promote cathodic polarization and suppress the growth of Cu films in the DC electrodeposition process. However, as the concentration of JGB increased to 20 mg/L, the twinning characteristics gradually disappeared.

**Figure 11:**
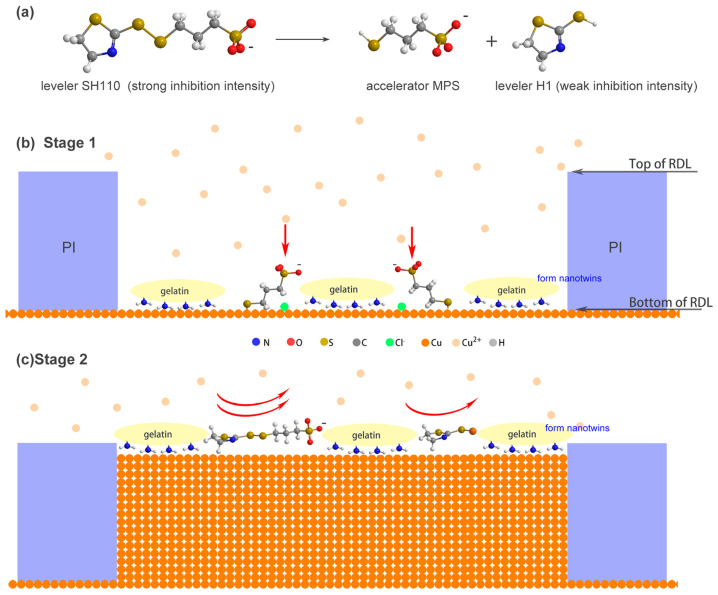
Schematic illustration of additive effect during electroplating process: (**a**) decomposition reaction of SH110, (**b**) copper deposition at the bottom of RDLs, (**c**) copper deposition at the top of RDLs [71]. Red arrows refer to themovement direction of Cu^2+^.

In 2023, Zhi-Quan Liu’s group [71] chose sodium thiazolinyl dithiopropane sulphonate (SH110) as a leveler to fabricate nt-Cu films and fill fine-pitch redistributed layers (RDLs). SH110 with a strong inhibition intensity could break down into the levelers of H1 and MPS during electroplating, as shown in Figure 11. SH110 could work with gelatin as a co-additive to grow nt-Cu in RDLs, and the mechanism of the process was analyzed with linear sweep voltammetry (LSV) and galvanostatic measurement (GM). As an accelerator, MPS worked at the bottom of the RDLs to promote Cu deposition, and gelatin was used to grow nanotwins without being affected. The SH110 and H1 suppressed the deposition at the top of the RDLs. However, as mentioned above, other levelers such as JGB could induce a detwinning effect with gelatin owing to the interaction and competition adsorption of the additive. Therefore, the interaction between the growth of nanotwins and the role of additives is still a mystery.

The development of the growth mechanism of nanotwins has great significance for guiding the application of nt-Cu in microelectronic packaging. Although many researchers have worked hard to find out the formation mechanism (as mentioned in Section 3.1 and Section 3.2), the root cause remains unknown due to its difficulty of observation. The key influencing factors are mainly categorized as pulse current (electric field), a high stirring rate (hydrodynamics field), and adequate additives (chemical field).

### 3.3. Electroplating nt-Cu Applied in Microelectronic Packaging

**Figure 12:**
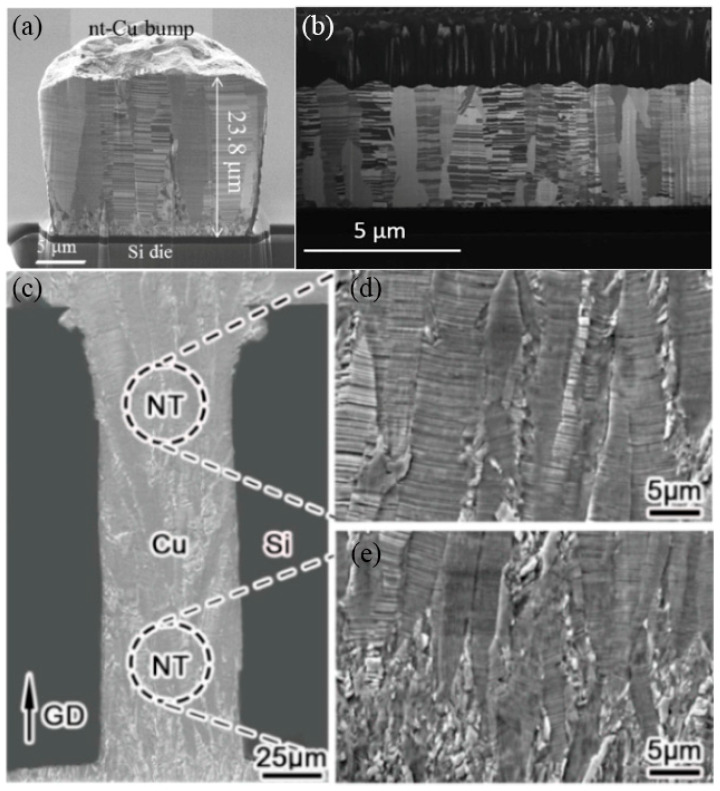
Cross-sectional FIB images of (**a**) electroplated nt-Cu bumps [24]. (**b**) nt-Cu RDLs along the long axis [26]. (**c**–**e**) The microstructure of an nt-Cu-filled through-silicon via (TSV) [25].

Modified electrolyte and electroplating processes of nt-Cu films have been applied in microelectronic packaging technology, such as UBM, CuPs, RDLs, and TSVs, as shown in Figure 12.

Luhua Xu and coworkers [72] first fabricated wafer Cu interconnection columns with fine grains and high-density nanotwins in opening sizes (15–100 μm) and depths (200–400 μm) using aspect-ratio-dependent electroplating. Later, Chih Chen’s group [21] fabricated [111] nt-Cu with patterned UBM with a 20 μm diameter in 2012, which introduced the unidirectional growth of (0001) Cu_6_Sn_5_ grains in solders. (111)-oriented nt-Cu was also fabricated as CuPs through DC electroplating in 2016 and 2018 [24,73]. The electrolyte used included a high-purity CuSO_4_ solution, proper surfactants, and 40 ppm of HCl, and a high stirring speed was essential during the electroplating. The deposition rate was 25 nm/s when the stirring rate was 1200 rpm.

In 2018, Zhi-Quan Liu’s group [25] first fabricated large-scale nt-Cu in TSVs using the DC electroplating process. The microstructure of the deposited Cu was <111>-oriented with columnar grains, and the nanotwin lamella was about 22 nm, as shown in Figure 12c–e. In 2021, M. L. Huang’s group [74,75] electroplated (111) nt-Cu films through UBM on common polycrystalline copper substrates using a CuSO_4_-based electroplating solution with suitable additives. Additionally in 2021, Zhi-Quan Liu’s group [69] developed a novel additive strategy to fill nt-Cu in 15/15 μm line/space RDL patterns. The coplanarity of the wafer lines and pads was lower than the industrial threshold of 8% for other patterns [76]. I-Hsin Tseng and coworkers [26] fabricated nt-Cu in RDLs with 800:10:5 μm (in length: width: height) and an extremely thin transition layer, as shown in Figure 12b. Currently, the preparation and application of nanotwinned copper have aroused some interest in large enterprises, such as Taiwan Semiconductor (TSMC), Advanced Semiconductor Engineering (ASE), and Lam Research, as inferred from their website news and patents.

Research on nt-Cu preparations and applications in the field of microelectronic packaging are summarized in Table 1. Previous research can provide a solid theoretical basis for the practical applications of nanotwinned copper. However, there are some challenges and limitations of nt-Cu in the fabrication technique and economic aspect. On the one hand, for the PED electroplating process, the electroplating time of nt-Cu is prolonged compared to normal Cu. On the other hand, for the DC electroplating process, the stability of additives for nt-Cu are worse than normal Cu. The monitoring of additive content is more difficult. Furthermore, a stabilizer is necessary for the mass production of nt-Cu, which could increase cost.

Above all, there are several issues that need to be addressed for fabrication before nt-Cu is applied in electronic packaging.

How can the transition layer at the bottom of nt-Cu films be eliminated and the microstructure regularity and properties be improved? It is important as the RDLs and UBM become thinner and thinner.

The growth mechanism of nanotwins in the DC electroplating process needs to be clarified. In addition, whether it can remain in agreement between the DC and PED processes remains unknown. It is important for the stable preparation of nanotwins in large areas, including 6-inch, 8-inch, and 12-inch wafers.

How can the filling ability of nt-Cu electrolytes be improved without influencing the microstructure of high-density nanotwins. Good filling ability is urgently needed for patterns such as RDL vias and TSVs.

There is not too much toxicity in the copper electroplating process, but some levelers containing nitrogen heterocyclic molecules have some toxicity, which needs to be noted in the subsequent waste processing after electroplating.

## 4. Reliability of nt-Cu in Electronic Packaging

Novel materials need thorough reliability evaluation before real application. In recent decades, the reliability evaluation of nt-Cu in UBM, CuPs, RDLs, and TSVs has been studied and reported on, successively. Therefore, in this chapter, we discuss the effect of nt-Cu on the improvement of reliability in practical applications, such as interfacial reaction layers (including UBM and CuPs), RDLs, and TSVs.

### 4.1. Interfacial Reaction Layers

The anisotropic microstructure of nt-Cu is one of the concerning issues for the applications of interfacial reaction layers in advanced packaging. Chih Chen’s group [87] fabricated Cu pillars in the nanoscale with various orientations to investigate the stress–relaxation behavior of nt-Cu. With the use of an in situ Picoindenter under constant strain and in situ TEM, dislocations disappeared quickly due to much shorter paths in nanopillars with twin orientations parallel to the axial direction of the pillars (NT-0°), which was different from that in Cu bulk or in Cu thin film. On the contrary, a long distance increased the dislocation density on the (111) plane by dislocations piled up in nanopillars. In addition, the nanopillars with a twin orientation vertical to the axial direction of the pillar (NT-90°) had a short path to the free surface, which changed the mechanical property from bulk nt-Cu. In conclusion, a dislocation-reduced region in the NT-0° nanopillar under constant strain caused worse stress–relaxation behavior than that of the NT-90° nanopillar that maintained a high dislocation density.

**Figure 13:**
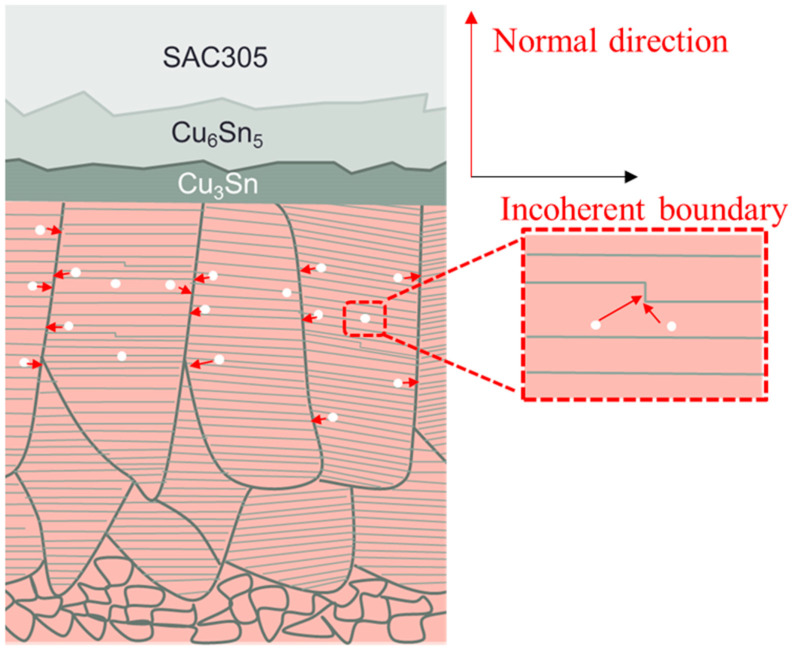
The schematic of vacancy sink sites in the bamboo structure of nt-Cu [89]. Red arrows represent the direction of vacancy migration.

The Kirkendall effect is another issue concerned in UBM and CuP applications. However, densely packed nanotwins in (111)-oriented Cu can eliminate the formation of Kirkendall voids in solder joint reactions [88]. In 2012, Chih Chen’s group [21] fabricated the unidirectional growth of a Cu_6_Sn_5_ IMC on (111)-oriented nt-Cu electroplated using the DC process. Very few Kirkendall voids could be seen on the Cu_3_Sn–nt-Cu interface during solid-state aging at 150 °C for 500 h. The explanation for this phenomenon was that the high density of TBs in nt-Cu films could serve as vacancy sinks during the aging reaction and, thus, the number of Kirkendall voids declined. Chih-Ming Chen’s group [89] fabricated nt-Cu with various crystal orientations that was thermally aged with Sn-rich solder at 200 °C. Interfaces with four kinds of microstructures, i.e., grain orientations of high (111), intermediate (111), high (110), and very high (110) Cu films, were compared. The Σ3 TB in (111)-oriented nt-Cu with a bamboo structure could effectively suppress the Kirkendall voids at the Sn-solder–Cu interface during thermal aging, as shown in Figure 13. The columnar GBs and dense TBs provided numerous vacancy sinks to reduce the possibility of void nucleation.

The growth behavior of intermetallic compounds (IMCs) has been reported as well. M. L. Huang’s group [75] examined in situ the microstructural evolution in (111)-oriented nt-Cu/Sn/polycrystalline Cu interconnects during aging at 300 °C for 10 s. The IMC growth rate of nt-Cu was almost six times faster than that of polycrystalline Cu, while the consumption–dissolution rate of nt-Cu was less than that of polycrystalline Cu. This anomalous phenomenon was explained by the concentration gradient control (CGC) interfacial reaction model, where the existence of high-density nanotwins decreased the surface energy and Cu atom diffusion from polycrystalline Cu/Sn to the nt-Cu–Sn interface. In addition, Zhi-Quan Liu’s group [90] found the self-healing of Kirkendall voids at the interface after thermal aging due to the fast diffusion rate of Cu atoms on the (111) plane.

Notably, void formation at the solder–Cu joints may also be induced by the existence of impurities [91,92]. Additives in the electrolyte can introduce impurities in Cu films to form voids during thermal aging. It has been found that additives can result in a higher level of impurity within Cu films and further affect the atomic deposition behavior of Cu. High-angle GBs are more prone to introduce impurities, while CTBs have fewer impurity adsorption sites due to less atomic mismatch. Hsuan Lee and coworkers [84] fabricated a Sn–nt-Cu joint with little impurity compared with a Sn–Cu joint. A higher shear strength was obtained in Sn–nt-Cu joints with little impurity. Therefore, the formation of Kirkendall voids is not only related to the microstructure of Cu, but is also related to impurities. Further studies are needed to prove the relationship between the nanotwin microstructure and the elimination of Kirkendall voids.

In addition to the influence on interfacial reactions, the influence on the mechanical strength of solder joints under thermal aging tests was also analyzed. I-Hsin Tseng and coworkers [93] electroplated a 3.8 μm nt-Cu film on a Si wafer substrate for thermal stress measurement from room temperature to 400 °C. The (111)-oriented nt-Cu began to transform to (200)-oriented Cu at 150 °C. The compressive stress that nt-Cu could withstand was 1.5 times greater than that of the randomly oriented Cu, which drove the anisotropic grain growth of nt-Cu. M. L. Huang’s group [74] fabricated (111)-oriented nt-Cu UBMs to investigate the IMCs and the shear strength of their solder joints after thermal aging. A (11–20)-preferred orientation roof-type Cu_6_Sn_5_ IMC formed at 300 °C, while a random-orientation scallop-type Cu_6_Sn_5_ IMC formed at 250 °C. Moreover, the average shear strength of solder joints soldered at 300 °C was 30% higher than that of those soldered at 250 °C.

### 4.2. Redistribution Layers (RDLs)

Yu-Jin Li and coworkers [34] confirmed potential applications of nt-Cu RDLs with a yield strength range of 300–700 MPa and an elongation rate range of 5–25%. The thermal strains of nt-Cu and regular Cu RDLs at various temperatures were analyzed by X-ray nanodiffraction [94]. In the results, the maximum stress of nt-Cu was 344.6 MPa at 434 K, which was 7.4% greater than that of the neighbor area, while the maximum stress of regular Cu was 363.8 MPa. The maximum thermal strain or stress appeared at the corner of the RDL where the line turned 90°, and the stress gradient in the corner might have caused the early failure in the thermal cyclic tests. Later, Yu-Bo Zhang and coworkers [95] also electroplated nt-Cu RDLs and achieved superior uniformity and flatness compared to three other commercial copper electrolytes.

Wafer warpage is caused by the diversity of materials used in RDLs and the complicacy of constitutive models during deformation restricting the development of RDLs. Gong Cheng and coworkers [79] suggested that introducing nanotwins in Cu RDLs relaxed great stress at the initial cooling and reduced the total thermal stress during the cooling process from 400 °C to ambient temperature, further reducing the wafer warpage introduced by Cu RDLs effectively.

**Figure 14:**
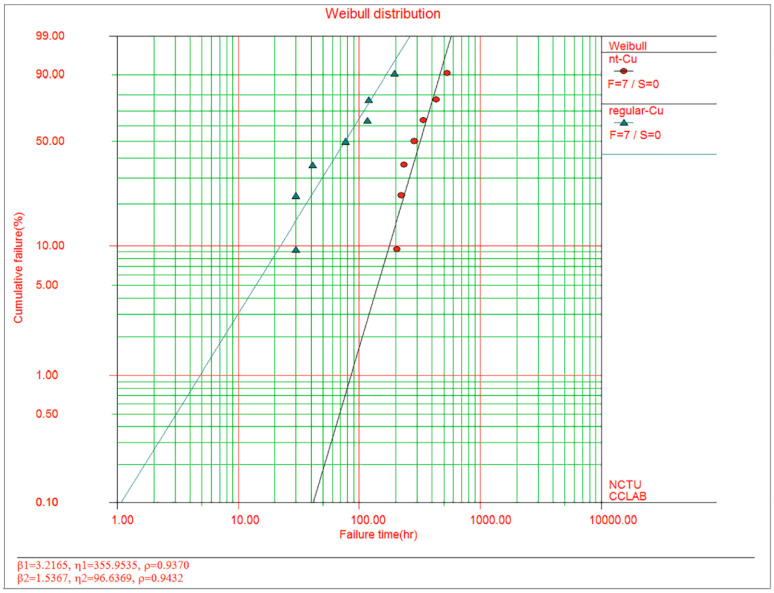
Plot of cumulative EM failure percentage against failure time for nt-Cu versus regular Cu RDLs. The t50% of nt-Cu RDLs was 320 h (the red circular points), whereas it was only 80 h for regular Cu RDLs (the green triangle points) [26].

In 2021, Tseng, I. Hsin and coworkers [26] analyzed the EM failure mechanisms of (111)-oriented nt-Cu and common Cu RDLs, as shown in Figure 14. The failure of RDLs could be attributed to the EM-induced voiding and oxidation, and the EM lifetime of nt-Cu RDLs was four-fold longer than that of common Cu. This long EM lifetime made possible the application of nt-Cu in fine-pitch advanced packaging. Superior EM resistance of nt-Cu was also proved by other studies [10].

### 4.3. Through-Silicon Vias (TSVs)

As early as 2018, Fu-Long Sun and coworkers [25] prepared columnar nt-Cu TSVs. TSVs were checked by nanoindentation from bottom to top, and the hardness was 2.34 GPa at the top and 2.63 GPa at the bottom. The small difference (0.29 GPa) between the top and the bottom sides of the nt-Cu TSVs verified the uniformity of the filled nt-Cu TSVs. Compared with the hardness for Cu TSVs without twins (1.73 GPa), the high mechanical properties of nt-Cu provided a framework to prevent wafer warpage during the later wafer-thinning process.

**Figure 15:**
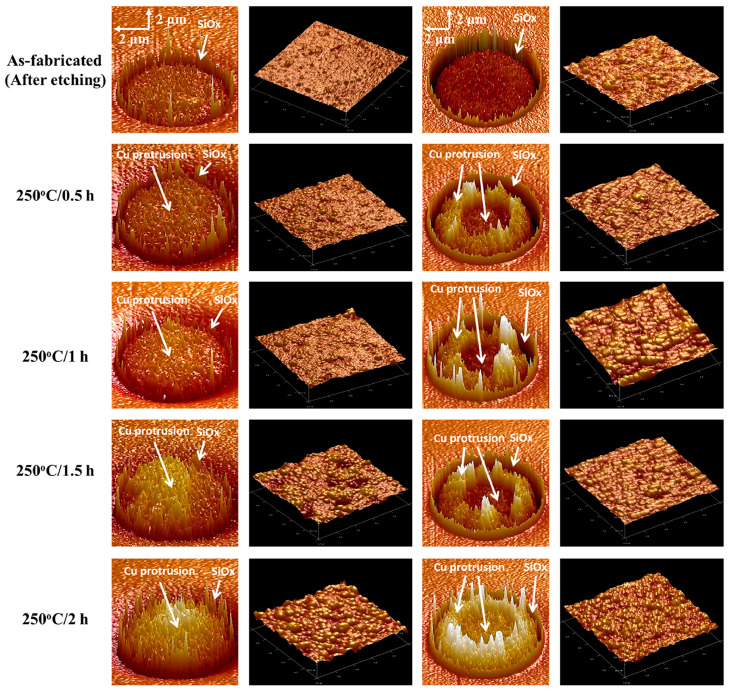
AFM 3D topography maps of normal Cu and nt-Cu TSVs during thermal annealing at 250 °C for 0.5, 1, 1.5, and 2 h. The Cu protrusion phenomenon was characterized through a 7 μm (x) × 12.5 μm (y) or 10 μm (x) × 12.5 μm (y) 3D topography map, and the grain morphology was investigated through a local 1 μm (x) × 1 μm (y) map [98].

Bongyoung Yoo’s group proposed [96,97] that nt-Cu fabricated with the PED process might effectively suppress the thermal extrusion of Cu TSVs. The energy supplied during the heat treatment could be consumed during the release of twins and strain fields in nt-Cu TSVs. Ting-Chun Lin and coworkers [98] inhibited detrimental Cu protrusion in TSVs by introducing highly (111)-oriented nt-Cu. The reason for the divergent protrusion behavior between the inner and outer regions in nt-Cu TSVs, as shown in Figure 15, was that there were two regions containing nt-Cu grains in the center and normal Cu grains near the side wall of the TSVs. Despite the presence of the normal Cu grains in nt-Cu TSVs, the existence of high-density nanotwins still raised the strengthening of TSVs, as well as resulting in a 70.3% decrement of the protrusion height during thermal annealing at 250 °C for 2 h.

### 4.4. Other Applications in Microelectronic Packaging

In addition to UBM, CuPs, RDLs, and TSVs, there have also been some applications reported on in recent years, such as reaction layers with Ag solder pastes [99], low-temperature Cu–Cu direct bonding, and Damascene vias [67]. Among them, low-temperature Cu–Cu direct bonding is the most hotly researched and has reaped wide attention.

Zhi-Quan Liu’s group [99] used nt-Cu as the substrate of a sinter, and the bonding strength of (111)-oriented nt-Cu with Ag paste was better than that of random-oriented Cu. The bonding strength of Cu paste on an nt-Cu film could be up to 53.7 MPa at the sintering condition of 300 °C, and the value on common Cu film was 31.3 MPa.

Traditional interconnect alloys, such as lead-free Sn-based alloy solders, has become unsuitable for the relatively low resistance of electromigration and thermomigration in 3D fine-pitch packaging. From this perspective, researchers have proposed Cu–Cu direct bonding [24,100]. It has been noticed that Cu–Cu bonding can be achieved at room temperature under ultrahigh vacuum conditions and 300 °C thermal compression over 30 min under normal vacuum conditions and that the latter can save a lot of time. However, conventional solders only need a welding temperature below 250 °C, so the key to the development of Cu–Cu bonds is to successfully realize the bonding under a normal vacuum below 250 °C. Jia-Juen Ong and coworkers [101] achieved low-temperature and low-pressure (111)-oriented nt-Cu–SiO_2_ hybrid bonding. The bonding temperature could be lowered to 200 °C with a pressure of 1.06 MPa, which had a very low specific contact resistance of 1.2 × 10^−9^ Ω/cm^2^ and excellent thermal stability up to 375 °C. The Cu–Cu direct bonding using (111)-oriented nt-Cu could be accounted for by a surface diffusion creep-assisted bonding mechanism [24,102]. Based on surface creep diffusion, there are four steps of Cu-to-Cu bonding: surface contact; thermal compression; void ripening; and boundary elimination. Two Cu surfaces integrate closely where plastic deformation may occur, and then they exert thermal compression to form twist-type GBs through surface creep where the surfaces hit each other.

Chih Chen’s group [103] studied the failure mechanisms of Cu–Cu bumps during TCT. More nt-Cu columnar grains remained compared with other crystalline grains under bonding. Anisotropic grain growth of nt-Cu film was observed at 250 and 300 °C in Cu-to-Cu joints [104]. The transformation from a (111)- into a (100)-preferred orientation driven by thermal strain energy improved the bonding strength of the joints. The shear strength performance of the Cu–Cu joints was influenced by the microstructure of the bonded interfaces [86]. The fracture mode of (111)-oriented Cu pillar–Cu pillar joint bonds was brittle at the temperatures of 200 °C/100 °C but ductile after bonded above 300 °C/100 °C, which was influenced by voids and the grain growth behavior of Cu at the bonded interface.

As a new kind of promising interconnected material, nt-Cu initially assisted solder reactions in film form and currently fills in shallow or deep vias. Extensive literature has proved that the reliability of packaging structures is improved due to the presence of nt-Cu, owing to its high strength, high surface diffusivity, high EM lifetime, and high thermal stability. Therefore, more and more researchers recognize the potential of nt-Cu as a semiconductor material, not only serving as a BEOL (back end of the line) material, but also as a FEOL (front end of the line) material in the future, such as for hybrid bonding and Damascene vias.

## 5. Conclusions

In summary, this paper reviewed the unique physical properties, the optimization of preparation, and the reliability evaluation of nt-Cu in the microelectronic packaging field. The superior physical properties of nt-Cu have caused nt-Cu materials to maintain a high level of research fervor. Secondly, the electroplating process and electrolytes of nt-Cu in recent decades were summarized. The growth mechanism of nanotwins and key influencing factors were discussed from the aspects of the electrical field, the hydrodynamics field, and the chemical field. Thirdly, nt-Cu has good performances in various applications of microelectronic packaging, such as UBM, CuPs, RDLs, TSVs, and Cu–Cu bonding. The mass production of nt-Cu can be anticipated in the near future. However, there are also many challenges to utilizing nt-Cu in microelectronic packaging before it can be mass-produced, including the following:

1. Enhancement of microstructure regularity and reduction of impurity concentration to ensure the good physical properties of nt-Cu under the trend of electronic packaging miniaturization.
2. Improvement of the filling performance of nt-Cu electrolytes for different patterns on wafers.
3. Evaluation of thorough reliabilities (force, thermal, and electrical aspects) for specific applications.

## Figures and Tables

**Table 1:** Summaries of electrolyte components and electroplating parameters for nt-Cu materials in the field of electronic packaging.

Reference	Electric Field	Environment	VMS	Additives	Nature of nt-Cu
Lei. Lu [11]	PED: t_on_ = 0.02 s; t_off_ = 2 s; J_peak_ = 0.5 A/cm^2^	pH~1 20 ± 1 °C	CuSO_4_	/	Film: The average grain size was about 400 nm and contained a high density of growth twins of the {111}/[112] type.
Madoka Hasegawa [59]	PED: −0.2 V or −0.6 V vs. SCE; t_on_ = 0.02 s; t_off_ = 1–4 s	standard three-electrode cell with a Pt mesh and a saturated calomel electrode	65 g/L CuSO_4_· 5H_2_O 1196 g/L H_2_SO_4_ 50 mg/L HCl	100 mg/L polyethylene glycol (Mw 4000) 10 mg/L 3-Mercapto-1-propanesulfonic acid sodium salt	Film: Horizontal nt-Cu obtained at −0.2 V vs. SCE, and twin spacing reduced with increasing t_off_; vertical nt-Cu obtained at −0.6 V vs. SCE.
Gong Cheng [77]	PED: J_peak_ = 100 mA/cm^2^; frequency = 2.5 Hz; J_avg_ = 1 ASD	pH = 1	80 g/L CuSO_4_ 50 ppm Cl^−^	/	Film: The average grain diameter was about 1.93 μm. The average twin lamella thickness was about 116.2 nm.
Xiaodei Zhan [52]	PED: J_avg_ = 2 ASD; γ = 30%	Stirring rate: 800 rpm	15–125 g/L CuSO_4_· 5H_2_O 184 g/L H_2_SO_4_ 10 ppm Cl^−^	/	Film: Twin thickness decrease with increase in the pulse frequency; a spiral feature of a hexagonal pyramid was found on the top of nt-Cu.
Mengya Dong [78]	PED: J_peak_ = 4 A/cm^2^; t_on_ = 20 ms; t_off_ = 1 s; 2000 cycles	pH = 1 25 °C	1.0 M CuSO_4_ NaCl	/	Cu micro-cone arrays with densely packed vertical nanotwins.
Gong Cheng [79]	PED: t_on_ = 4 ms; t_off_ = 196 ms; J_avg_ = 2 ASD	pH = 1	CuSO_4_ 80 g/L 50 ppm Cl^−^	/	RDL: The thickness of Cu foils was about 7 μm. The clear twin lamellae averaged 116.2 nm and were perpendicular to the film surface.
Sanghyun Jin [80]	PED: J_peak_ = 50–200 mA/cm^2^; t_on_ = 0.02 s; t_off_ = 0.65 s	pH = 0.8	0.8 M CuSO_4_ 50 ppm HCl	1 mL/L S-Additive A (Samsung Electronics Co.) 5 mL/L S-Additive B (Samsung Electronics Co.)	TSV: The aspect ratio of via was 9.17 (diameter = 6 μm, depth = 55 μm; v-shaped TSV filling; win spacing was about several tens of nanometers.
Yu-Xi Wang [67]	PED: J_peak_ = 60 ASD; t_on_ = 0.02 s; t_off_ = 0.5–4 s	Stirring rate: 300 rpm 25 ± 1 °C	50–100 g/L CuSO_4_· 5H_2_O H_2_SO_4_ 50 ppm Cl^−^	PEG 0–40 ppm MPS	Damascene via: The via was about 1.4 μm deep,1.4 μm wide at the bottom, and 1.9 μm wide at the top; the aspect ratio was about 1:1.
Kuan-Ju Chen [44]	Periodic-reverse (PR) wave forms: J_peak_/J_zvg_ = 8/8 or 8/4 ASD; t_on_/t_rev_ = 40/1–40/6	/	196 g/L CuSO_4_ 40 ppm Cl^−^	4 mL/L organic additive	Film: Highly <111>-oriented columnar nt-Cu with thin transition layers.
I-Hsin Tseng [26]	Periodic-reverse electroplating: J_on-time_ = 4 ASD, t_on_ = 40 ms J_off-time_ = −1 ASD, t_off_ = 4 ms	/	0.8 M Cu^2+^ in CuSO_4_ 40 ppm HCl 100 g/L H_2_SO_4_	additives	RDL: The length was 800 μm, the height was 5 μm, and the width was 10 μm with a denser nt-Cu with a thin area of fine grain at the bottom.
K. N. Tu’s group [72,81,82,83]	Aspect-ratio-dependent electroplating 30 h	/	Cu 40 g/L Sulfuric acid 150 g/L Chloride 50 mg/L	Leveler 15 mL/L Brightener 10 mL/L	Through-wafer Cu interconnection: Twin lamellar width was ~20 nm.
Hsuan Lee [84]	DC: 0.34 ASD	28 °C	0.88 M CuSO_4_· 5H_2_O 0.54 M H_2_SO_4_ 60 ppm NaCl	PEG (8000 g/mol)	Film: Slender columnar nanotwins with Σ3 TBs (60° rotation at <111>) perpendicular to the substrate.
Chih-Han Tseng [64]	DC: 5–11 ASD 8 min	Stirring rate: 1200 rpm	0.8 M Cu^2+^ 50–110 g/L H_2_SO_4_ 80 ppm Cl^−^	4000 ppm ECD-107A	Film: (111)-oriented columnar nt-Cu grains. Twin thickness increased with current density.
Chun-Cheng Lin [85]	DC: 400 mA/cm^2^	12 ± 0.2 °C	1.08 M CuSO_4_· 5H_2_O 25 mM H_2_SO_4_ 0.7 mM NaCl	60–300 ppm MPS	Film: Thickness of twin lamella ranged from 10–240 nm; large columnar grains composed of highly ordered twin lamellae with a v-shaped grain boundary.
Yu Bai [61]	DC: 1–5 ASD	Stirring rate: 200 rpm	195 g/L CuSO_4_· 5H_2_O 13 mL/L H_2_SO_4_ 40 ppm NaCl	commercial additive	Film: Vertical nt-Cu with a (220) texture.
Fu-Long Sun [66]	DC: 30 mA/cm^2^	Stirring rate: 300 rpm	0.8 mol/L Cu^2+^ 3–17 mL/L H_2_SO_4_ 40 ppm NaCl	organic additive	Film: Equiaxed crystals of small size (grain size of about 100 nm) near the bottom substrate, columnar twin-free grains in the middle (lateral size of about 6.8 μm), and columnar nanotwinned grains near the top surface (lateral size of about 7.9 μm).
K. N. Tu’ group [24,86,87]	DC: 40–100 mA/cm^2^	Stirring rate: 1200 rpm	CuSO_4_ solution 40 ppm HCl	surfactants	CuP: Densely-packed (111) nanotwins aligned vertically in the growth direction.
Hsiang-Yao Hsiao [21]	DC: 80 mA/cm^2^	Stirring rate: 600–1200 rpm	CuSO_4_ 40 ppm HCl	proper surfactants	UBM: 20 μm thick (111)-oriented columnar nt-Cu.
Tao-Chi Liu [60]	DC: 20–70 mA/cm^2^	Stirring rate: 400–1500 rpm	0.8 M Cu^2+^ in CuSO_4_ solution with 40 ppm HCl	surfactants	UBM: 20 μm thick Cu pad with a 100 μm diameter; the columnar grains were [111]-riented. The average grain size was 3.4 ± 0.7 μm.
Jing Huang [71]	DC: 30 mA/cm^2^	Stirring rate: 300–900 rpm 25 ± 2 °C	120–200 g/L CuSO_4_· 5H_2_O 3–80 mL/L H_2_SO_4_ 30–100 ppm NaCl	0–100 ppm gelatin 0–20 ppm SH110	RDL: The width and pitch of the RDLs were both 15 μm; equiaxed fine grains near the seed layer and columnar grains at the top of the RDLs.
Fu-Long Sun [25]	DC: 30 mA/cm^2^	Stirring rate: 300 rpm	75 g/L CuSO_4_ 3 mg/L H_2_SO_4_	5–70 mg/L gelatin	TSV: Diameter of 30~50 μm and aspect ratio of ~4. The average twin thickness was about 22 nm.

## Data Availability

All data generated or analyzed during this study are included in this published article.
